# Data on taxonomic status and phylogenetic relationship of tits

**DOI:** 10.1016/j.dib.2016.11.079

**Published:** 2016-11-28

**Authors:** Xue-Juan Li, Li-Liang Lin, Ai-Ming Cui, Jie Bai, Xiao-Yang Wang, Chao Xin, Zhen Zhang, Chao Yang, Rui-Rui Gao, Yuan Huang, Fu-Min Lei

**Affiliations:** a Co-Innovation Center for Qinba regions’ sustainable development, College of Life Sciences, Shaanxi Normal University, Xi’an 710062, China; b Key Laboratory of the Zoological Systematics and Evolution, Institute of Zoology, The Chinese Academy of Sciences, Beijing 100101, China; c Shaanxi Institute of Zoology, Xi’an 710032, China

## Abstract

The data in this paper are related to the research article entitled “Taxonomic status and phylogenetic relationship of tits based on mitogenomes and nuclear segments” (X.J. Li et al., 2016) [1]. The mitochondrial genomes and nuclear segments of tits were sequenced to analyze mitochondrial characteristics and phylogeny. In the data, the analyzed results are presented. The data holds the resulting files of mitochondrial characteristics, heterogeneity, best schemes, and trees.

**Specifications Table**

**Table:** 

Subject area	Biology, Genetics and Genomics
More specific subject area	Phylogenetics and Phylogenomics
Type of data	Figures, Tables, Trees
How data was acquired	The analyses of A+T contents, conserved site percentages and P-distances, were obtained in MEGA 4.1 [2]. Sequences were aligned in Muscle [3]. The best schemes were analyzed in Partitionfinder v1.1.1 [4]. The heterogeneity was inferred with AliGROOVE [5]. The gene trees based on six datasets (one mitochondrial dataset and five nuclear segments) were constructed in RAxML 7.0.3 [6]. A species tree was obtained with employing these gene trees in ASTRAL [7].
Data format	Analyzed
Experimental factors	The RY-coding method was employed for the third sites of protein-coding genes, while nuclear dataset was divided into different parts (exons and introns).
Experimental features	The phylogeny employed the best schemes inferred by PartionFinder v1.1.1 [4]. A species tree was obtained by employing gene trees in ASTRAL [7].
Data source location	Shaanxi Normal University
Data accessibility	Data is with this article

**Value of the data**

• The provided files of comparative mitochondrial characteristics of tits can be valuable to further summarize.
• The files of phylogenetic relationships would help to further study the phylogeny of tits and even Passeriformes.
• The provided ‘.tree’ files can be directly used to compare with other results.

## 1 Data

**Fig. 1:**
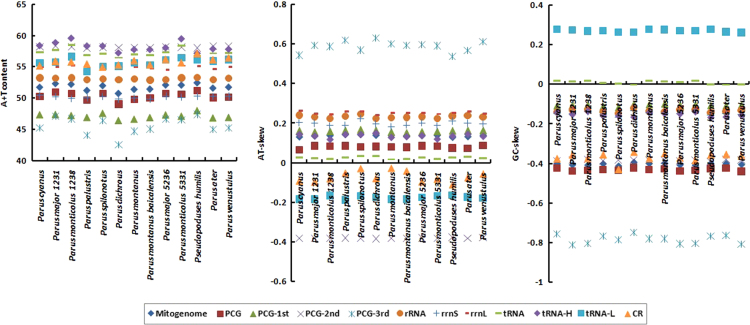
Nucleotide compositions of different mitochondrial partitions in 10 tits species. *Note:* AT-skew ([A−T]/[A+T]), GC-skew ([G−C]/[G+C]), PCG-1st (the first codon positions of protein-coding genes), PCG-2nd (the second codon positions of protein-coding genes), PCG-3rd (the third codon positions of protein-coding genes), tRNA-H (the tRNA genes on H-strand), tRNA-L (the tRNA genes on L-strand).

**Fig. 2:**
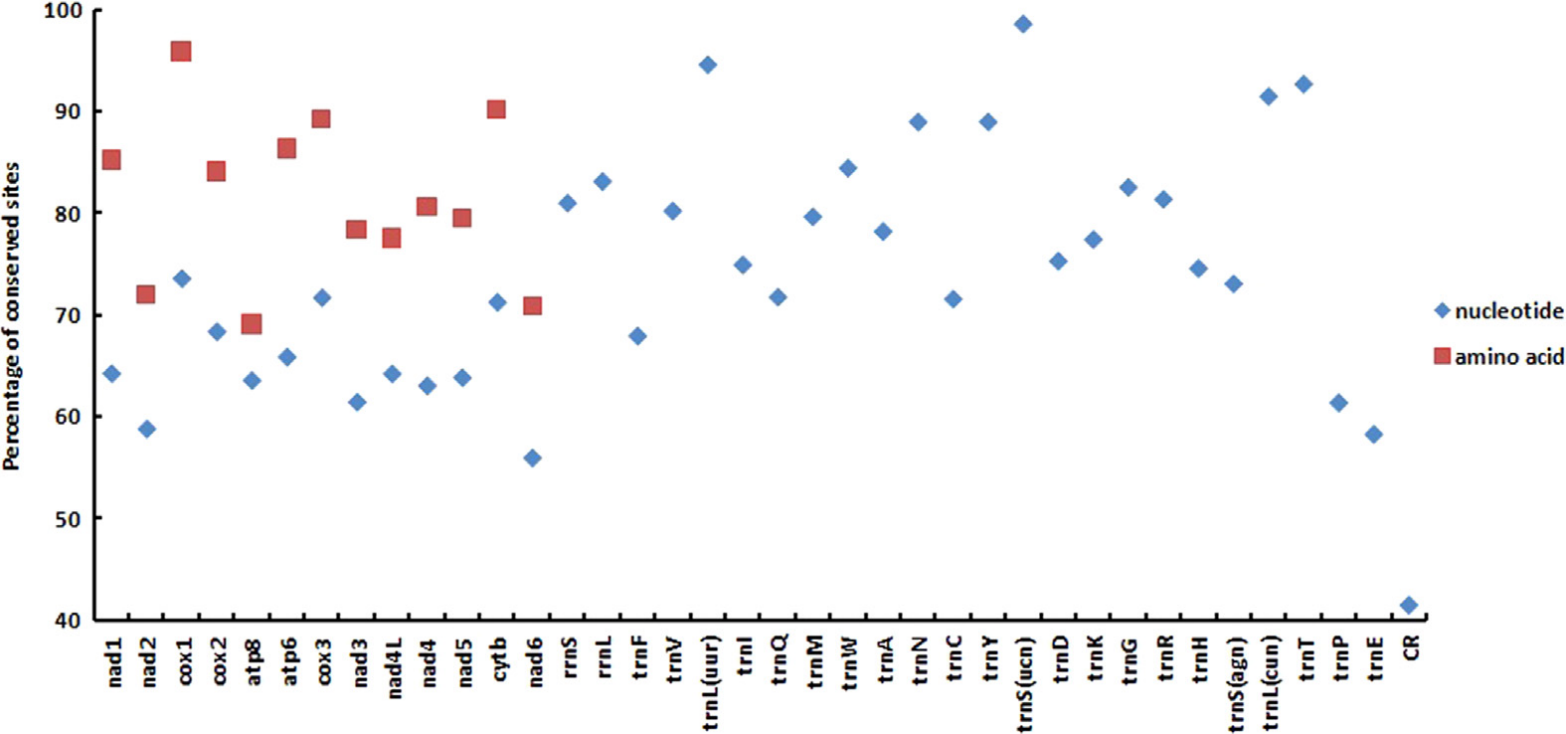
Conserved site percentages of mitochondrial genes among 10 tits species.

**Fig. 3:**
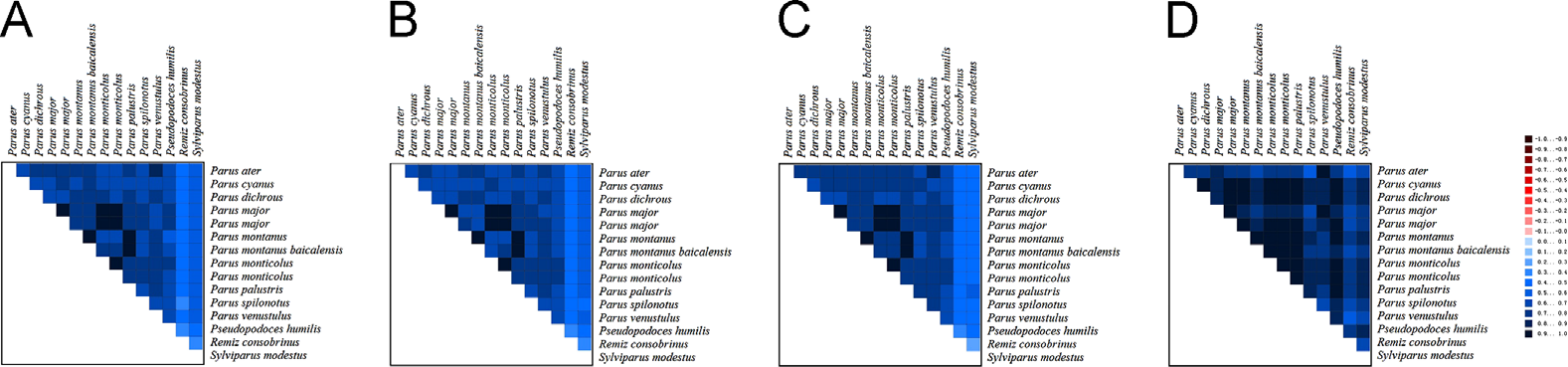
The heterogeneity analyzed by AliGROOVE. *Note:* The heterogeneity continuously decreased from −1 (red coloring) to +1 (blue coloring). A: the first and second codon positions of protein-coding genes, B: protein-coding genes with the third codon positions not using RY-coding method, C: mitochondrial genome with the third codon positions not using RY-coding method, D: nuclear segments dataset. (For interpretation of the references to color in this figure legend, the reader is referred to the web version of this article).

**Fig. 4:**
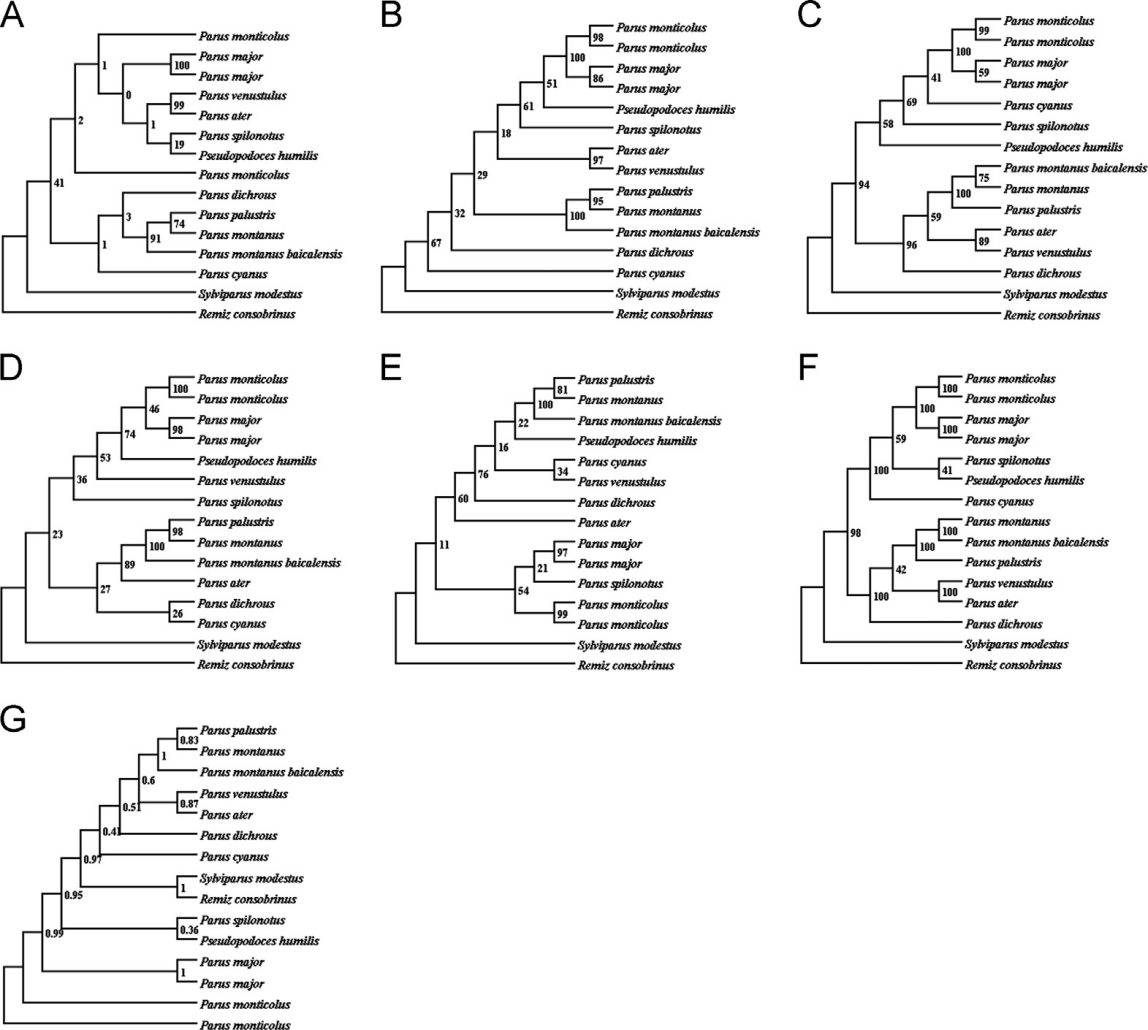
The gene trees and species tree analyzed by using ASTRAL. *Note:* The gene trees (A–F) were constructed based on maximum likelihood method. A: MOS; B: FGB; C: ALDOB; D: PCBD1; E: CALB1; F: mitochondrial genome; G: species tree.

In the data, Fig. 1, Fig. 2 show base compositions and conserved site percentages of tits, respectively. Fig. 3 is the result of heterogeneity. Fig. 4 shows gene trees and a species tree. Table 1 describes the taxonomic samples. Table 2 lists the primer sequences. Table 3 is the P-distance based on mitochondrial dataset. Table 4 shows the best schemes.

## 2 Experimental design, materials and methods

This study sampled 13 individuals of tits by using *Sylviparus modestus* and *Remiz consobrinus* as outgroups. Each gene was aligned in Muscle [3] independently. The mitochondrial characteristics, including A+T contents, conserved site percentages and P-distances, were analyzed by using MEGA 4.1 [2], and the results can be found in Fig. 1, Fig. 2 and Table 3, respectively.

Four datasets, A: the first and second sites of protein-coding genes, B: protein-coding genes with the third sites not employing RY-coding method, C: 37 mitochondrial genes with the third sites of protein-coding genes not using RY-coding method plus one control region, D: five nuclear segments, were used to analyze the heterogeneity in AliGROOVE [5], and the results can be found in Fig. 3. The best schemes were analyzed by using Partitionfinder v1.1.1 [4], and the results were in Table 4. The gene trees in Fig. 4 were constructed by using RAxML 7.0.3 [6], employing 1000 replications, and these results were used to construct a species tree by using ASTRAL [7].

## Figures and Tables

**Fig S2:**
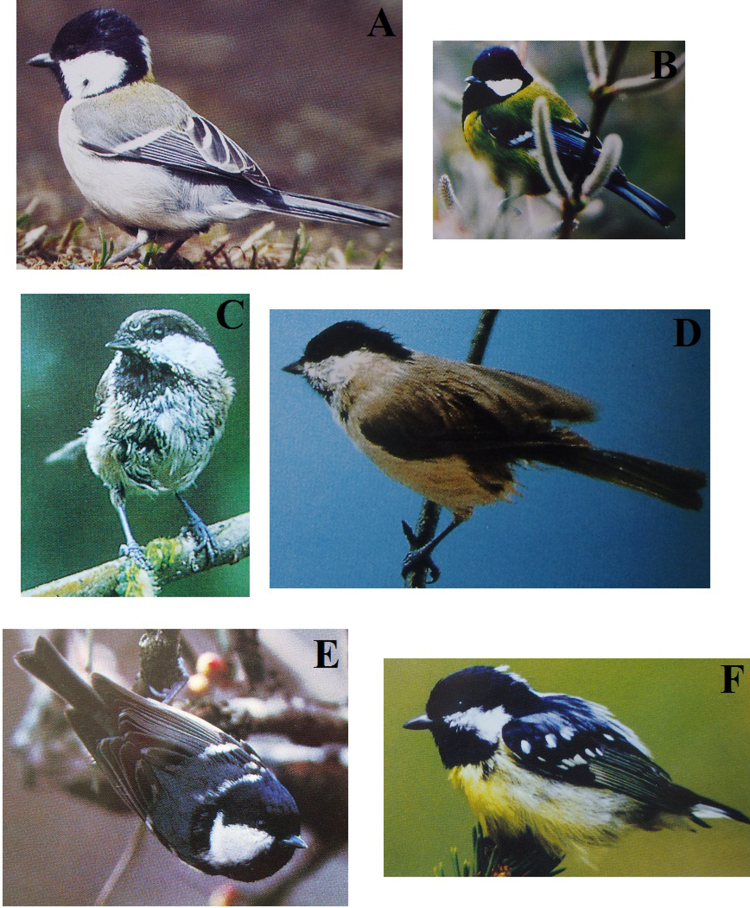
The figures of six tits species represented three sister groups. Note: A: *P. major*; B: *P. monticolus*; C: *P. montanus*; D: *P. palustris*; E: *P. ater*; F: *P. venustulu*.

**Table 1:** Taxonomic samples in the study.

Family	Genus	Species and subspecies	Sample locality/source	GenBank accession Nos.					
				Mitogenome	MOS	FGB	ALDOB	PCBD1	CALB1
Paridae	*Parus*	*Parus major*	Beach forestry centre, Zhouqu County, Gansu Province	KX388473	KX388398	KX388413	KX388428	KX388443	KX388458
		*Parus major*	Baihualing, Gaoligongshan, Yunnan Province	KX388480	KX388405	KX388420	KX388435	KX388450	KX388465
		*Parus monticolus*	Beach forestry centre, Zhouqu County, Gansu Province	KX388474	KX388399	KX388414	KX388429	KX388444	KX388459
		*Parus monticolus*	Dahaoping, Gaoligongshan, Yunnan Province	KX388481	KX388406	KX388421	KX388436	KX388451	KX388466
	*Poecile*	*Parus montanus*	Liancheng, Yongdeng County, Gansu Province	KX388478	KX388403	KX388418	KX388433	KX388448	KX388463
		*Parus montanus baicalensis*	Maoershan, Shangzhi City, Heilongjiang Province	KX388479	KX388404	KX388419	KX388434	KX388449	KX388464
		*Parus palustris*	Beach forestry centre, Zhouqu County, Gansu Province	KX388475	KX388400	KX388415	KX388430	KX388445	KX388460
	*Cyanistes*	*Parus cyanus*	Kizil, Baicheng County, Xinjiang	KX388472	KX388397	KX388412	KX388427	KX388442	KX388457
	*Machlolophus*	*Parus spilonotus*	Longqishan Nature Reserve, Fujian Province	KX388476	KX388401	KX388416	KX388431	KX388446	KX388461
	*Lophophanes*	*Parus dichrous*	Sanguanmiao, Shaanxi Province	KX388477	KX388402	KX388417	KX388432	KX388447	KX388462
	*Periparus*	*Parus ater*	Wen County, Gansu Province	NC_026223	KX388408	KX388423	KX388438	KX388453	KX388468
	*Pardaliparus*	*Parus venustulus*	Yangxin County, Huangshi City, Hubei Province	NC_026701	KX388410	KX388425	KX388440	KX388455	KX388470
	*Pseudopodoces*	*Pseudopodoces humilis*	Bird Island, Qinghai Lake, Qinghai Province	KP001174	KX388407	KX388422	KX388437	KX388452	KX388467
	*Sylviparus*	*Sylviparus modestus*	Luding County, Sichuan Province	NC_026793	KX388409	KX388424	KX388439	KX388454	KX388469
									
Remizidae	*Remiz*	*Remiz consobrinus*	Xinxing Town, Panjin City, Liaoning Province	NC_021641	KX388411	KX388426	KX388441	KX388456	KX388471

**Table 2:** The primers used in this study.

Name	Sequence(5′–3′)	Name	Sequence(5′–3′)
L1263b^a^	AAAGCATRRCACTGAA	H10343b	TGGGCTCATGTGACKGTRACKCC
H1859b	TCGATTACAGAACAGGCTCCTCTA	L10236b	TTCTGAGCMTTCTTCCAYTCMAG
L1754b	TGGGATTAGATACCCCACTATG	H10884b^a^	GGGTCRAAWCCRCATTCGTATGG
H2294b	TTTCAGGTGTAAGCTGAATGCTT	L10635b^a^	CACCACTTYGGCTTYGARGCAGC
L2260b^a^	CAAGGTAAGTGTACCGGAAGGTG	H11837b	ARGGTKGCTTCRAATGCRATRTARAA
H2891b^a^	TGATGGCTGCTTRARGGCCCAC	L11458b	TCYACCCGAACYCACGGCTCMGA
L2725b	CGAGCCGGGTGATAGCTGG	H12344b	CTATGTGGCTKACKGAKGAGTAKGC
H3292b	TGATTGCGCTACCTTTGCACGG	L12156b	CCHAAAGCMCACGTAGAAGCMCC
L3218b	CGACTGTTTACCAAAAACATAGCC	H13047b	CTTTTACTTGGATTTGCACCAA
H3784b	CGGTCTGAACTCAGATCACG	L13040b^a^	ATCCAATGGTCTTAGGARCCA
L3722b	GGTTTACGACCTCGATGTTGG	H13563b^a^	TGRAGGGCDGCRGTGTTRGC
H4170b	CCYACRATRTTTGGGCCTTTKCG	L13525b	GMTGAGAAGGRGTAGGAATCATATC
L3803b	CTACGTGATCTGAGTTCAGACCG	H14127b	CCTATTTTTCGRATGTCYTGTTC
H4644b	TCRAATGGGGCRCGRTTTGTYTC	L14080b	TCAACYCACGCATTCTTYAARGC
L4500b	GTAGCCCAAACAATCTCMTAYGARG	H15049b	GTGTCTGCTGTGTAGTGYATDGC
H5201b	CCATCATTTTCGGGGTATGG	L14770b	TMGGMCCAGAAGGAYTVGC
L5143b	GAACCTRCACWARAGRGATCAAAAC	H15295b	CCTCAGAATGATATTTGKCCTCAKGG
H5766b	GGAKGAGAAGGCTAKGATTTTTCG	L14996b	AACATCTCADCHTGATGAAACTTYGG
L5758b	GGRGGMTGAATAGGMCTAAACCARAC	H15646b	GGYGTGAARTTTTCTGGRTCTCC
H6681b^a^	GGTATAGGGTDCCRATGTCTTTRTG	L15413b	GGWGGATTYTCAGTAGACAACCC
L6615b^a^	CCTCTGTAAAAAGGACTACAGCC	H16064b^a^	CTTCAATCTTTGGYTTACAAGACC
H7122b	GCTGTTGTRATGAAGTTGATDGCYCC	L15725b^a^	AAACCHGAATGATACTTCCTMTTYGC
L7036b	GGAACAGGATGAACYGTNTACCC	H1530b^a^	GGTGGCTGGCACARGATTTACC
H7548b	GTRGCGGATGTRAAGTATGCTCG	CMOSF	GCCTGGTGCTCCATCGACTGG
L7525b	GTNTGAGCMCACCACATRTTYAC	CMOSR	GCAAATGAGTAGATGTCTGCT
H8121b	GGGCAGCCGTGRATTCATTC	FIB4F	CTGTAATATCCCGGTGGTTTCAGG
L7987b	TCAGACTACCCAGAYGCCTAYAC	FIB4R	ATTTCAGATGTTTCACCTCCCTTTC
H8628b	TCGTAGGWTCAGTATCATTGRTGNCC	AldB6F	GAGCCAGAAGTCTTACCTGAYGG
L8386b	GCYTCATCMCCYATCATAGAAGA	AldB7R	CAGCTGTCACCATGTTNGG
H9235b	TCGAAGAAGCTTAGGTTCATGGTCA	DCOH3F	AGGCCTGGCTTCATGAC
L8929b	GGMCAATGCTCAGAAATYTGYGG	DCOH4R	GATAAACCYGTGCARTCYTGGGTGCT
H9726b	AGRTGKCCTGCTGTNAGRTTNGC	Cal9F	AGGGTGTCAARATGTGTGSGAAAGA
L9700b	GAAACAACAAGCCTACTHATYCGHCC	Cal11R	GTANAGCTTCCCTCCATCNGACAA

a Means the primers used in LA-PCR.

**Table 3:** The P-distance based on mitogenome dataset.

Species													Genus							
*Parus cyanus*													*Cyanistes*							
*Parus major*	0.085												*Parus*	0.085						
*Parus monticolus*	0.084	0.051											*Poecile*	0.092	0.088					
*Parus palustris*	0.092	0.087	0.088										*Machlolophus*	0.090	0.083	0.094				
*Parus spilonotus*	0.090	0.084	0.083	0.094									*Lophophanes*	0.096	0.089	0.080	0.096			
*Parus dichrous*	0.096	0.089	0.089	0.080	0.096								*Pseudopodoces*	0.096	0.091	0.099	0.094	0.101		
*Parus montanus*	0.091	0.088	0.088	0.038	0.093	0.080							*Periparus*	0.093	0.089	0.079	0.094	0.082	0.102	
*Parus montanus baicalensis*	0.092	0.089	0.088	0.038	0.094	0.080	0.021						*Pardaliparus*	0.092	0.087	0.077	0.092	0.080	0.099	0.071
*Parus major*	0.086	0.021	0.052	0.087	0.084	0.090	0.088	0.088												
*Parus monticolus*	0.085	0.052	0.010	0.088	0.083	0.089	0.088	0.088	0.053											
*Pseudopodoces humilis*	0.096	0.091	0.090	0.097	0.094	0.101	0.100	0.099	0.091	0.091										
*Parus ater*	0.093	0.090	0.089	0.080	0.094	0.082	0.078	0.079	0.090	0.089	0.102									
*Parus venustulus*	0.092	0.089	0.086	0.077	0.092	0.080	0.077	0.076	0.088	0.086	0.099	0.071								

**Table 4:** Best schemes analyzed by PartitionFinder.

Dataset	Subset	Subset partitions	Optimal model
Protein-coding genes	P1	atp6_pos1, nad1_pos1, nad2_pos1, nad3_pos1, nad4L_pos1, nad4_pos1, nad5_pos1	GTR+I+G
	P2	atp6_pos2, atp8_pos2, cox3_pos2, cox2_pos2, cox1_pos2, cytb_pos2, nad1_pos2, nad2_pos2, nad3_pos2, nad4L_pos2, nad4_pos2, nad5_pos2	GTR+I+G
	P3	atp6_pos3, atp8_pos1, atp8_pos3, cox3_pos3, cox2_pos3, cox1_pos3, cytb_pos3, nad1_pos3, nad2_pos3, nad3_pos3, nad4L_pos3, nad4_pos3	GTR+G
	P4	cox3_pos1, cox2_pos1, cox1_pos1, cytb_pos1	GTR+I+G
	P5	nad5_pos3, nad6_pos3	GTR+G
	P6	nad6_pos1, nad6_pos2	GTR+G
			
Mitogenomes	P1	rrnS, rrnL, atp6_pos1, nad1_pos1, nad2_pos1, nad3_pos1, nad4L_pos1, nad4_pos1, nad5_pos1, trnR, trnD, trnG, trnH, trnI, trnK, trnM, trnF, trnS(agy), trnW, trnV	GTR+I+G
	P2	atp6_pos2, atp8_pos2, cox3_pos2, cox2_pos2, cox1_pos2, cytb_pos2, nad1_pos2, nad2_pos2, nad3_pos2, nad4L_pos2, nad4_pos2, nad5_pos2	GTR+I+G
	P3	atp6_pos3, atp8_pos3, cox3_pos3, cox2_pos3, cox1_pos3, cytb_pos3, D_loop, nad1_pos3, nad2_pos3, nad3_pos3, nad4L_pos3, nad4_pos3	GTR+I+G
	P4	cox3_pos1, cox2_pos1, cox1_pos1, cytb_pos1, trnN, trnL(uur), trnL(cun), trnS(ucn), trnT, trnY	GTR+I+G
	P5	atp8_pos1, nad5_pos3, nad6_pos3	GTR+I+G
	P6	nad6_pos1, nad6_pos2, trnA, trnC, trnQ, trnE, trnP	GTR+I+G
			
Nuclear segments	P1	ALDOB_exon, CALB1_exon, MOS_exon, PCBD1_exon, PCBD1_intron, FGB_exon	GTR+I+G
	P2	ALDOB_intron, CALB1_intron, FGB_intron	GTR+G

*Note:* Pos1, pos2, and pos3 indicate the first, second and third codon positions of protein-coding genes in mitogenomes, respectively.

## Appendix A Supplementary material

Supplementary material Supplementary Fig 1. The phylogenetic hypothesis of tits.

..

Supplementary material Supplementary file 1. The tree files of six gene trees and one species tree, corresponding to Fig. 4. Note: A: gene tree of MOS; B: gene tree of FGB; C: gene tree of ALDOB; D: gene tree of PCBD1; E: gene tree of CALB1; F: gene tree of mitochondrial genome; G: species tree.
