# Supplementary figures and images for: Data on taxonomic status and phylogenetic relationship of tits
Source: Data Brief. 2016 Nov 28;10:390–7. doi: 10.1016/j.dib.2016.11.079 (PMC5192249; doi:10.1016/j.dib.2016.11.079)

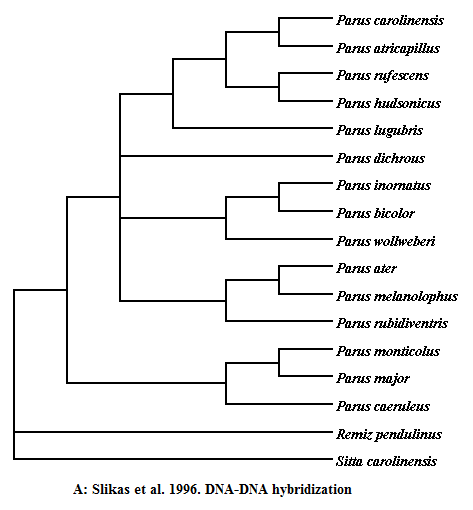

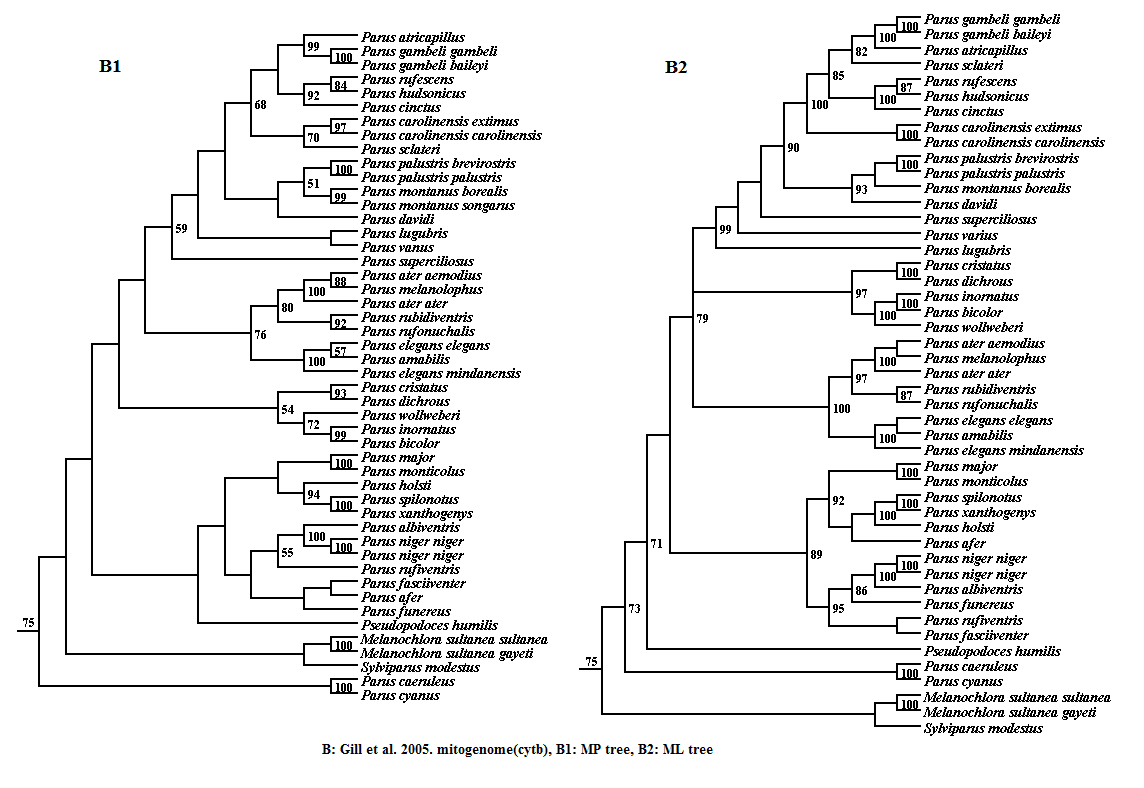


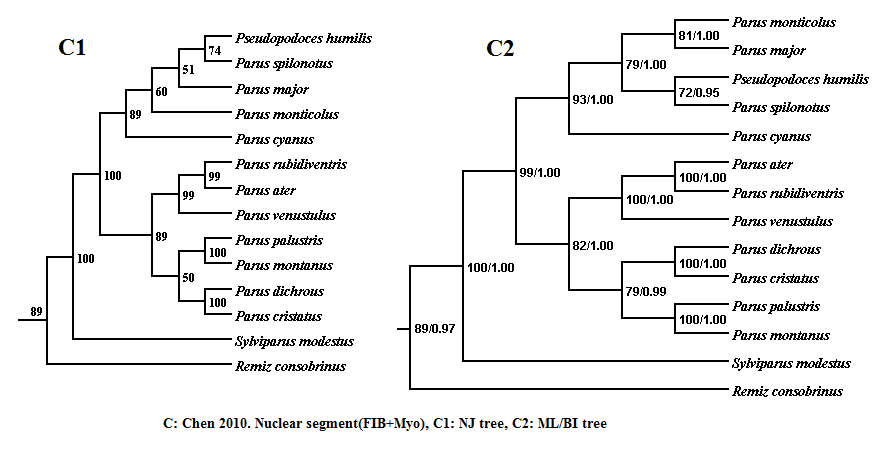

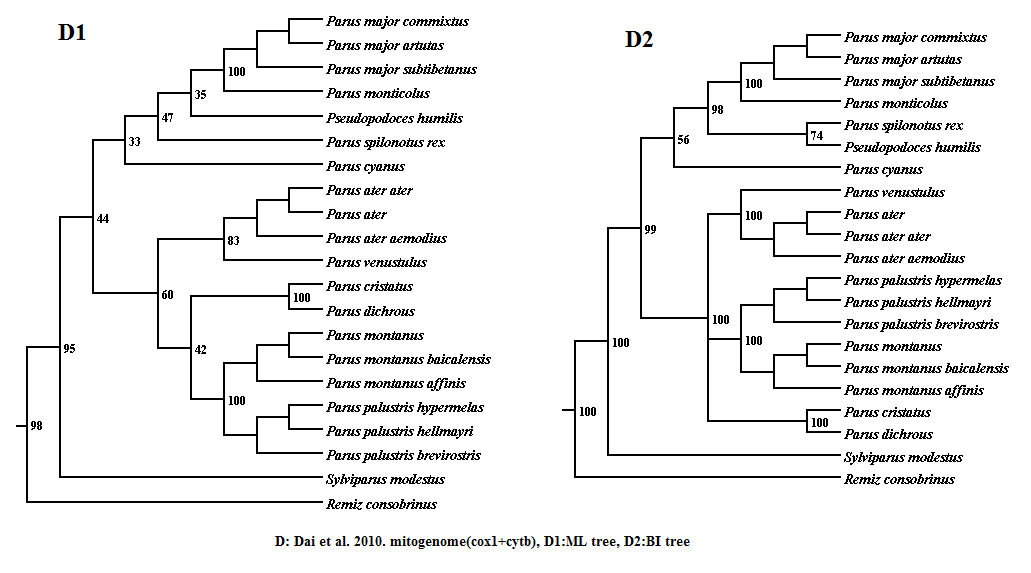


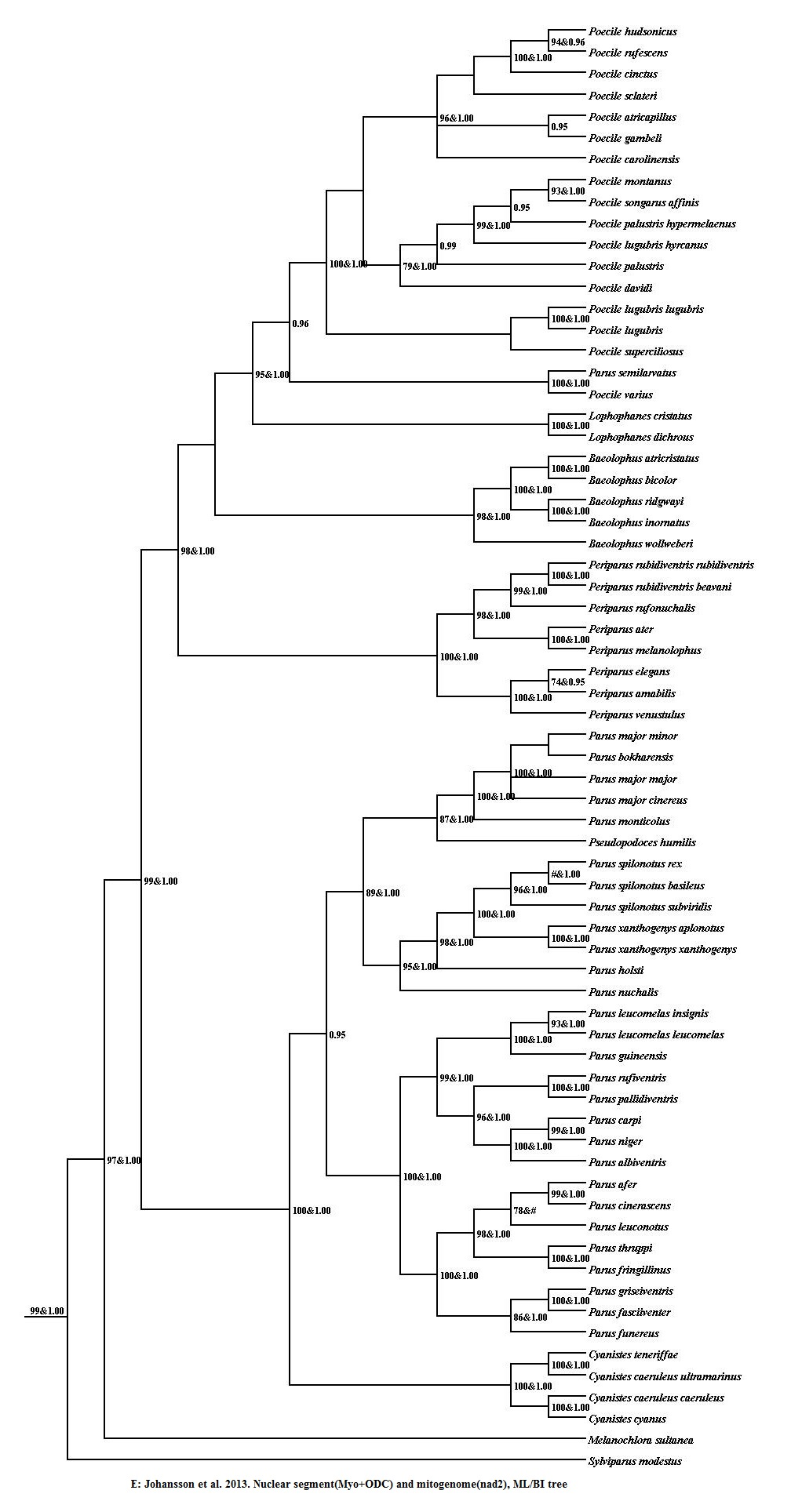


**Supplementary Fig. 1.** The phylogenetic hypothesis of tits.

Supplement: Supplementary file 2 — Supplementary material Supplementary Fig 1. The phylogenetic hypothesis of tits. [file mmc2.doc]
